# Mechanical unfolding of spectrin reveals a super-exponential dependence of unfolding rate on force

**DOI:** 10.1038/s41598-019-46525-w

**Published:** 2019-07-31

**Authors:** J. P. Renn, S. Bhattacharyya, H. Bai, C. He, H. Li, A. F. Oberhauser, J. F. Marko, D. E. Makarov, A. Matouschek

**Affiliations:** 1 Department of Molecular Biosciences, Northwestern University, Evanston, IL 60208 USA; 2 Department of Physics and Astronomy, Northwestern University, Evanston, IL 60208 USA; 3 Department of Chemistry, University of British Columbia, Vancouver, BC V6T 1Z1 Canada; 4 Department of Neuroscience and Cell Biology, Sealy Center for Structural Biology & Molecular Biophysics, University of Texas Medical Branch, Galveston, TX 77555 USA; 5 Department of Chemistry, The University of Texas at Austin, Austin, TX 78712 USA; 6 Oden Institute for Computational Engineering and Sciences, The University of Texas at Austin, Austin, TX 78712 USA; 7 Department of Molecular Biosciences, The University of Texas at Austin, Austin, TX 78712 USA

**Keywords:** Deformation dynamics, Polymer chemistry

## Abstract

We investigated the mechanical unfolding of single spectrin molecules over a broad range of loading rates and thus unfolding forces by combining magnetic tweezers with atomic force microscopy. We find that the mean unfolding force increases logarithmically with loading rate at low loading rates, but the increase slows at loading rates above 1pN/s. This behavior indicates an unfolding rate that increases exponentially with the applied force at low forces, as expected on the basis of one-dimensional models of protein unfolding. At higher forces, however, the increase of the unfolding rate with the force becomes faster than exponential, which may indicate anti-Hammond behavior where the structures of the folded and transition states become more different as their free energies become more similar. Such behavior is rarely observed and can be explained by either a change in the unfolding pathway or as a reflection of a multidimensional energy landscape of proteins under force.

## Introduction

Single molecule force spectroscopy is a well-established approach to probing force-induced protein denaturation^1–3^. Atomic force microscopy (AFM) is well suited for studying the mechanical unfolding of proteins such as I27, which display high mechanical stability requiring forces in the 100–300 pN range to unfold on typical experimental timescales^4–8^. Other proteins exhibit much lower mechanical stability and can be unfolded by forces in the range of 10 pN, which is arguably more typical of mechanochemical processes *in vivo*. Spectrin, which was the subject of recent AFM pulling studies^9–11^, is a case in point. Spectrin is part of the cytoskeletal scaffolding network that gives eukaryotic cells their shape^12^. Each spectrin protein consists of multiple repeats of an α-helical 3-helix bundle domain, and each repeat is connected to the next by an α-helical linker^12^. Spectrin unfolds at much lower forces than I27, and the mean unfolding force of spectrin is either independent^10^ or weakly dependent^9,11^ on the pulling speed, in contrast to the much stronger dependence found for I27^4,13^. More precise quantification of the pulling speed dependence of the unfolding force for spectrin was, however, impeded by the fact that low forces, and correspondingly low pulling rates, are difficult to explore with most current AFM instruments. Exploration of the low force/low loading rate regime is valuable because the loading rates *in vivo* are thought to be slower than those used in AFM unfolding experiments^14,15^.

Here, we combined magnetic tweezers with AFM to characterize how spectrin unfolds over a wide range of loading rates, including low loading rates not usually accessible by AFM. In the low loading rate regime, we find a stronger dependence of the unfolding force on loading rate than observed in the AFM unfolding assays employing high loading rates. This behavior can be indicative of an “anti-Hammond” effect where the unfolding transition state moves away from the folded state as the force is increased^16–18^. The rate at which the mean unfolding force increases with the loading rate *r* (i.e., the increase in the applied force per unit time) is related to the activation length Δx (i.e., the distance between the folded state and the transition state along the pulling coordinate *x*) through the relation^19,20^:

$$d\langle F\rangle /d\,\mathrm{ln}\,r\approx {k}_{B}T/{\rm{\Delta }}x.$$

Consequently, weaker pulling speed dependence at higher forces suggests that the activation length increases with the increasing force, meaning that the transition state moves further away from the folded state. This observation is interesting because it cannot be captured by simple one-dimensional models; indeed, most common theories of force-induced unfolding view it as diffusive dynamics in a one-dimensional potential and predict the activation length to decrease with increasing force. Anti-Hammond behavior is a signature of multidimensionality of the underlying free energy landscape^17,18,21,22^.

## Results

### Measuring spectrin unfolding and refolding at low loading rate

We used a previously developed magnetic tweezers setup^23,24^ to investigate the unfolding behavior of repeats 12 to 16 of the human β-spectrin protein^12^, following the methodology established to investigate the unfolding of filamin A and vinculin proteins at loading rates varying from 0.16 pN/s to 16 pN/s^14,23,25,26^.

To attach the protein to two surfaces, we fused a SNAP domain (New England Biolabs) to the N-terminus of the spectrin fragment and a 23 amino acid long biotinylation peptide^27^ to its C-terminus. The protein was expressed in *E. coli*, purified and biotinylated. The N-terminal SNAP domain allowed us to attach the protein to a glass slide modified with benzylguanine, and the biotinylated C-terminus could be picked up with a streptavidin coated magnetic bead (Figs SI 1–3; see Methods for details). We generated forces on the protein-bead complex by a magnet below the stage holding the glass slide. The magnet was under the control of a stepper motor that could move it toward or away from the stage to increase or decrease the force acting on the bead. The position and the fluctuations of the beads were monitored using a light microscope connected to a CCD camera (for details of the setup see^28^). The distance of the tethered bead from the surface was calculated by comparing the height of the bead to a reference bead on the glass surface and the force exerted on the bead was calculated from the thermal fluctuations of the bead as described previously^23,24,29,30^.

**Figure 1:**
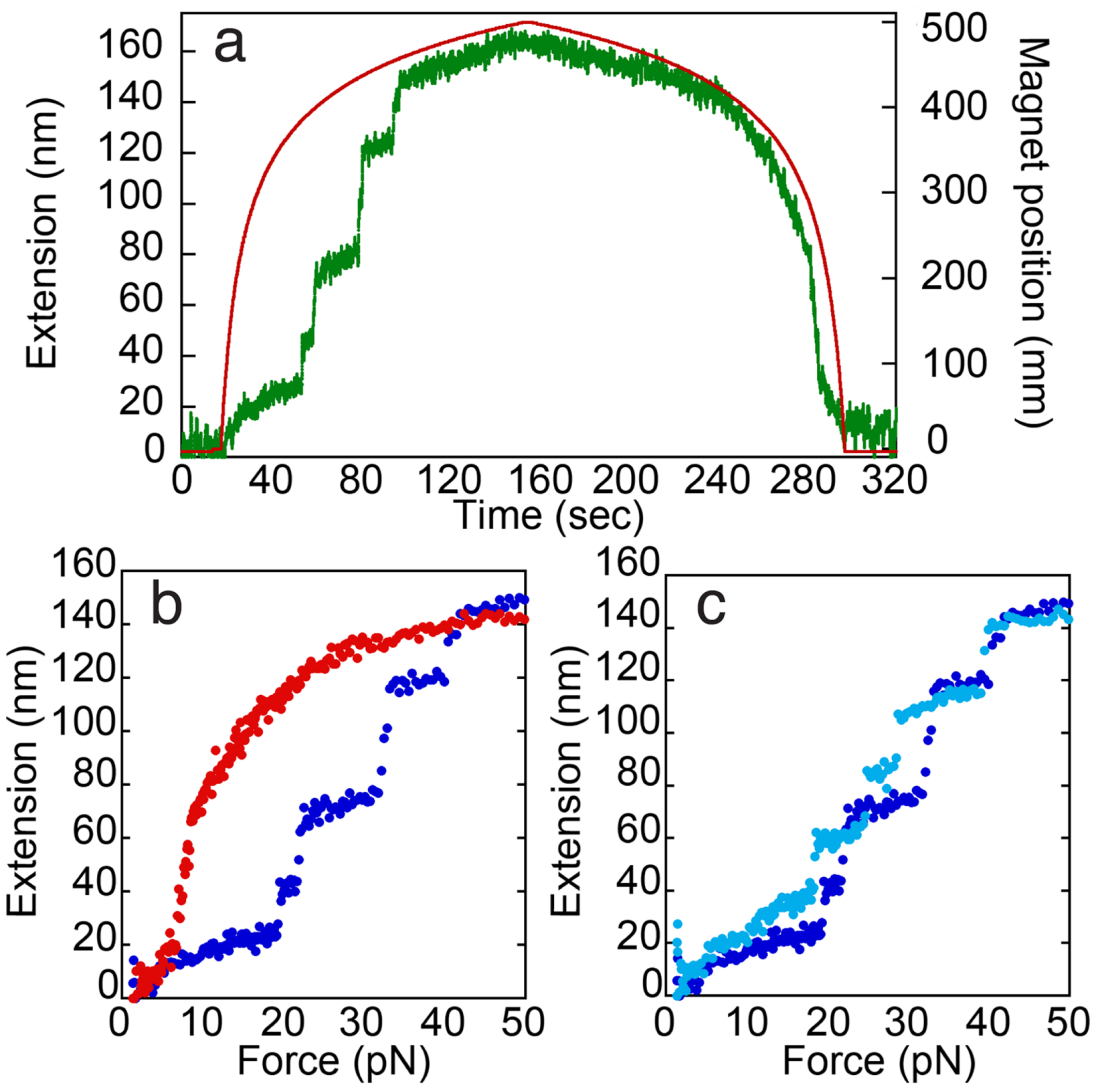
Force extension curves for spectrin unfolding by magnetic tweezers. (**a**) A representative trace of the extension (z-position of the beads) and force vs. time data for a typical spectrin pulling experiment. The data were smoothed using a sliding average of window of 10 ms. The magnet is moved relative to the microscope slide at a preprogrammed pattern to exert the desired linear force loading rate on a magnetic bead and the bead position is monitored. After the desired maximum force was reached to unfold the protein, the magnet was retracted at the desired rate and refolding was monitored. (**b**) Hysteresis is observed between unfolding (blue) and refolding (red). To construct the force vs. extension curve of the unfolding and refolding process the position at each force is averaged and plotted against the force. (**c**) Force vs. extension data for 2 subsequent unfolding cycles (dark blue first, light blue second) overlay suggesting that the tethered protein refolds properly, and attachment is specific.

In our initial experiments, we unfolded the spectrin protein by moving the magnet towards the surface such that the force exerted on the protein increased at a constant rate of *r* = 0.5 pN/s (Fig. 1A). As the force was increased, the protein was extended gradually, but the extension was interrupted by sharp step-like increases in its length. The gradually changing segments of the protein’s force-extension curve were well described by the wormlike chain (WLC) model^14^ suggesting that they resulted from the entropic elasticity of the unfolded parts of the polypeptide chain. We interpreted the sharp steps in the extension as the sudden unfolding of spectrin domains (Fig. 1).

This conclusion was supported by the calculation of the contour length released in each step (performed as described in ref.^14^), which matched the contour length of one or two spectrin domains (Fig. 1b). The total contour length released in an entire pulling cycle added up to that of five unfolded spectrin domains, which is the total number of spectrin domains in the construct (Fig. SI 4).

To characterize the refolding process, we unfolded a spectrin construct as described above, paused at a high force to allow the protein to reach equilibrium, and then retracted the magnet at the same speed as during unfolding but in the opposite direction until it returned to the starting position (Fig. 1a). The return trace did not overlay with the unfolding trace and lacked its distinct extension steps (Fig. 1b).

The hysteresis exhibited by the unfolding and refolding curves suggests that the extension/retraction cycle is a nonequilibrium process. To test whether the retraction arm of the experiment returns the protein to the native state, we unfolded the same tether a second time at the same loading rate. The second force vs. extension profile superimposed well with the profile from the first extension (Fig. 1c), although the individual transitions occurred at different positions as expected for stochastic events in a single molecule. We conclude that spectrin was unfolded reproducibly in the experiment. Generally, more than five unfolding and refolding cycles could be performed on a single protein before the protein no longer refolded and failed to return to its original extension.

### Measuring spectrin unfolding at different loading rates

**Figure 2:**
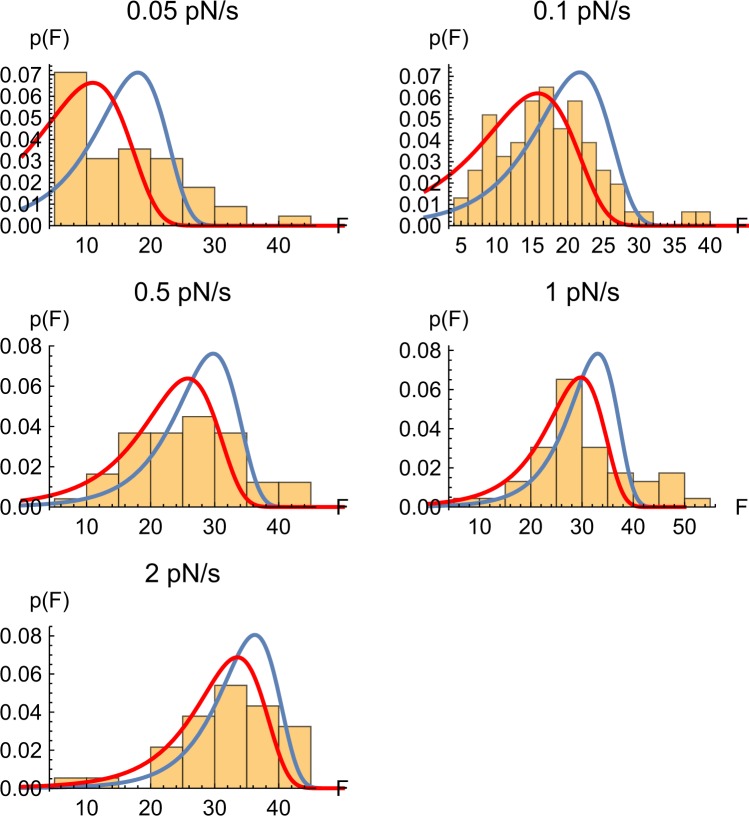
Experimental distributions of the unfolding force at different loading rates. Graphs plot the normalized number of unfolding events observed at the indicated force range at each loading rate = 0.05 pN s^−1^ (n = 41), 0.1 pN s^−1^ (n = 77), 0.5 pN s^−1^ (n = 49), 1 pN s^−1^ (n = 47) and 2 pN s^−1^ (n = 38). Blue and red lines are the distributions predicted by Eqs 1 and 3 with the parameters estimated from Eq. 2 (blue) and from the positions of the distribution peaks (red) as described in the text. The units of the force are pN and those of *p*(*F*) are 1/pN.

For most proteins that have been characterized by single molecule force spectroscopy, the mean unfolding force increases logarithmically with the loading rate, which is consistent with theoretical expectations^31^. Spectrin appeared to be an exception with its mean unfolding force either weakly dependent on or independent of the loading rate^9–11^. To test whether this behavior is a manifestation of a fundamental difference between spectrin and other proteins, we studied spectrin unfolding at five different loading rates *r*: 0.05, 0.1, 0.5, 1, and 2 pN/s. For each of these, we performed at least 13 pulls on three different tethers to analyze at least 37 transitions per loading rate (see supplementary methods for details). The histograms of the unfolding forces observed at each loading rate are shown in Fig. 2.

Our analysis only included the unfolding traces in which the total contour length released upon a complete extension added up to the unfolding of five spectrin domains. The remaining curves presumably originated from beads that were tethered to the glass surface by misfolded spectrin proteins or by several spectrin molecules simultaneously and were not included in the analysis.

To interpret the measured distributions of the unfolding force (Fig. 2) and their dependence on the loading rate, we adopted the common model that views protein unfolding as a first-order kinetic process with a rate coefficient *k*(*F*) that increases as the pulling force *F* applied to the protein’s termini is increased. The physical reason for this acceleration in the unfolding kinetics is that the force lowers the barrier to unfolding by performing mechanical work on the protein^32^. If the force dependence of the unfolding rate coefficient *k*(*F*) is known, then the mean unfolding force as a function of the loading rate *r* can be estimated^33^; moreover, the probability distribution of the force at which unfolding occurs *P*(*F*) can also be calculated. Specifically, the probability density *P*(*t*) of an unfolding step occurring at time *t* and thus having the magnitude *F* = *rt* (where *r* = *dF*/*dt* is the constant loading rate) is described by the equation^32^

$$P(t)=k(rt)\exp [-\,{\int }_{0}^{t}k(rt)dt]$$

Or, equivalently, the probability distribution *P*(*F*) of the unfolding force is given by

1 $$P(F)=P(t)dt/dF={r}^{-1}k(F)\exp [-{r}^{-1}{\int }_{0}^{F}dFk(F)]$$

In principle, Eq. 1 can be inverted to obtain *k*(*F*) from a distribution *P*(*F*) obtained at any specified value of the loading rate *r* using the formula^33^:

1′ $$k(F)=rP(F)/{\int }_{F}^{\infty }dF^{\prime} P(F^{\prime} )$$

**Figure 3:**
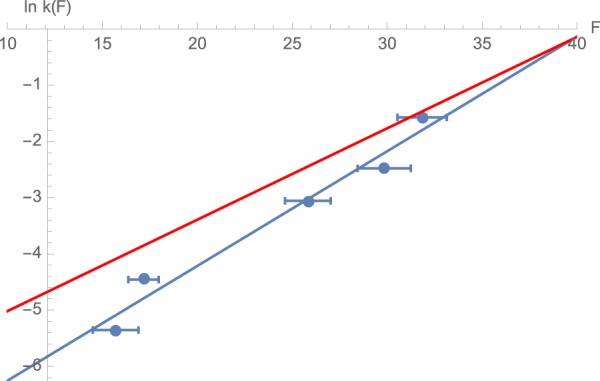
Estimated unfolding rate constants at different forces. The graph plots the unfolding rates estimated from Eq. 2 (blue circles) at the different unfolding forces. The lines show fits of the data to different models relating the unfolding rate of a protein to the applied force as described in Eq. 3 (blue line, parameters of Model A). Red line shows a different fit of *k(F*) using the same functional dependence (Eq. 3) but based on the position of the peaks of the distributions (Model B – see text for details.)

Unfortunately, incomplete sampling and the noise in the experimentally determined distributions *P*(*F*) renders this procedure inapplicable to our data. For this reason, we use an approximation to Eq. (1′) proposed by Dudko *et al*.^33^, which only requires the first and the second moments of the distribution rather than *P*(*F*) itself. This approximation is obtained by replacing *P*(*F*) with a Gaussian distribution and leads to the following result:

2 $$k(\langle F\rangle )\approx r{[\frac{\pi }{2}(\langle {F}^{2}\rangle -{\langle F\rangle }^{2})]}^{-1/2},$$

where $\langle F\rangle ={\int }_{0}^{\infty }FP(F)dF$ and $\langle {F}^{2}\rangle ={\int }_{0}^{\infty }{F}^{2}P(F)dF$ are the first and the second moments of the force distribution. The approximation states that the unfolding rate measured at the mean value of the force at which unfolding occurs at each loading rate is related to the variance of the force distribution. Applying Eq. 2 to the distribution of unfolding forces observed at each of the loading rates (Fig. 2) allowed us to estimate *k*(*F*) for different unfolding forces (*F*) (Fig. 3). Note, however, that the statistics of the measured unfolding forces did not appear to be very well described by Gaussian distributions, especially at the lowest loading rates (Fig. 2), making the assumptions underlying Eq. 2 questionable in our case. We will discuss this discrepancy further below.

As seen from Fig. 3, the logarithm of *k*(*F*) is well approximated as a linear function of the force *F*, or, equivalently, the force dependence of the unfolding rate is exponential,

3 $$k(F)={k}_{0}{e}^{aF}$$

This formula has a simple physical interpretation as the Arrhenius law,

$$k(F)=A{e}^{-\frac{{\rm{\Delta }}G(F)}{{k}_{B}T}}$$

written in terms of a force-dependent free energy barrier for unfolding Δ*G*(*F*).

The simplest model for this force dependence assumes that force lowers the barrier by the amount of work exerted on the molecule when it moves from the initial (i.e., native) state to the transition state. This work equals *F*Δ*x*, where Δ*x* is the activation length equal to the change in the protein extension between the folded and the transition states. Thus, the free energy barrier depends on the force according to the linear relationship:

4 $${\rm{\Delta }}G(F)\simeq {\rm{\Delta }}G(0)-F{\rm{\Delta }}x,$$

leading to an exponential force dependence, $k(F)=k(0){e}^{\frac{F{\rm{\Delta }}x}{{k}_{B}T}}$, which was originally proposed by Eyring to develop a molecular model of viscous friction^34^, used extensively to explain kinetic phenomena in fracture mechanics^35^, and later introduced by Bell into the field of biophysics^36^. We will be referring to this result as the Eyring-Zhurkov-Bell (EZB or simply Bell) formula. Comparing (4) and (3) one finds

5 $$a=\frac{{\rm{\Delta }}x}{{k}_{B}T}$$

Linear fit of force dependence ln*k*(*F*) estimated from Eqs 2 and 4, we estimate the activation length to be Δ*x* ≈ 0.84 nm, and ${k}_{0}=k(0)\approx 2.5\times {10}^{-4}\,{s}^{-1}$ (blue line in Fig. 3). We will refer to this set of parameters collectively as Model A.

At the same time, the shapes of the measured unfolding force distributions disagree significantly with the expected distributions calculated using these parameters for *k*_0_ and Δ*x* and Eqs 1 and 3 (Blue lines in Fig. 2). Since both the predicted and the experimental force distributions are significantly asymmetric, the assumption of a Gaussian distribution underlying Eq. 2 is violated. It is then instructive to fit the same data to the model described by Eq. 3 in a different way, namely by relating the maximum *F*_*m*_ of the distribution *P*(*F*), instead of the mean force 〈*F*〉, to the loading rate *r*. This relationship can be obtained by setting the derivative of Eq. 1 with respect to the force to zero, yielding the equation

6 $$\begin{array}{rcl}{\frac{d\mathrm{ln}k}{dF}|}_{F={F}_{m}} & = & {r}^{-1}k({F}_{m})\end{array},$$

Substituting Eq. 3 into Eq. 6 and using Eq. (5) leads to

7 $${F}_{m}={a}^{-1}\,\mathrm{ln}\,a+{a}^{-1}\,\mathrm{ln}(r/{k}_{0})=\frac{{k}_{B}T}{{\rm{\Delta }}x}\,\mathrm{ln}\,\frac{{\rm{\Delta }}x}{{k}_{B}T}+\frac{{k}_{B}T}{{\rm{\Delta }}x}\,\mathrm{ln}\,r-\frac{{k}_{B}T}{{\rm{\Delta }}x}\,\mathrm{ln}\,{k}_{0}$$

**Figure 4:**
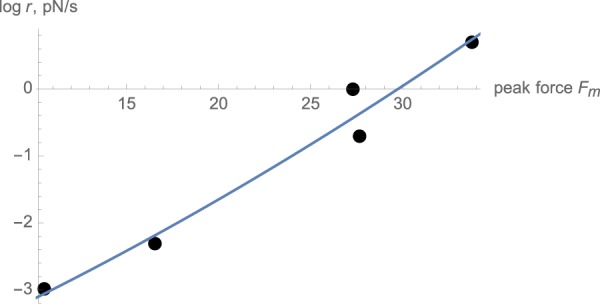
The relationship between the loading rate and the estimated peak of the unfolding force distribution. The solid line shows a fit of the loading rate, viewed as a function of the peak force, with Eq. 7. (Model B).

In other words, the peak force depends on the loading rate logarithmically. To estimate the peak force *F*_*m*_ from the measured force distributions at the different loading rates (Fig. 2), we applied a Gaussian kernel to smooth the raw data. Fitting the resulting relationship between *F*_*m*_ and the loading rate *r* with Eq. 7 (Fig. 4), one obtains somewhat different values for the model parameters: Δ*x* ≈ 0.67 nm, and *k*_0_ ≈ 1.6 × 10^−3^ *s*^−1^. As seen from Fig. 4, indeed, the dependence of the mean unfolding force on the loading rate is close to logarithmic, as predicted by Eq. 7. This set of parameters (which we will be referring to as Model B) provides a better fit for the shapes and the peak locations of the force distributions (Fig. 2, compare model B fit represented by red line to model A represented by blue line). However, model B predicts the mean unfolding force somewhat less accurately than Model A (Fig. 3, compare model B shown as the red line and model A shown as the blue line). Despite the discrepancy between the two models, both predict similar values of the activation length Δ*x*.

Although the EZB theory provides a reasonably good description of our magnetic tweezers data, the logarithmic loading rate dependence of the mean unfolding force we observe here is at odds with the much weaker dependence observed in earlier AFM studies of spectrin^10,11^, which were performed at much higher loading rates. Since those studies used somewhat different spectrin variants, we have carried out our own AFM measurements to investigate how mechanical response of spectrin depends on the loading rate and/or the experimental technique.

### AFM unfolding of spectrin at high loading rates

**Figure 5:**
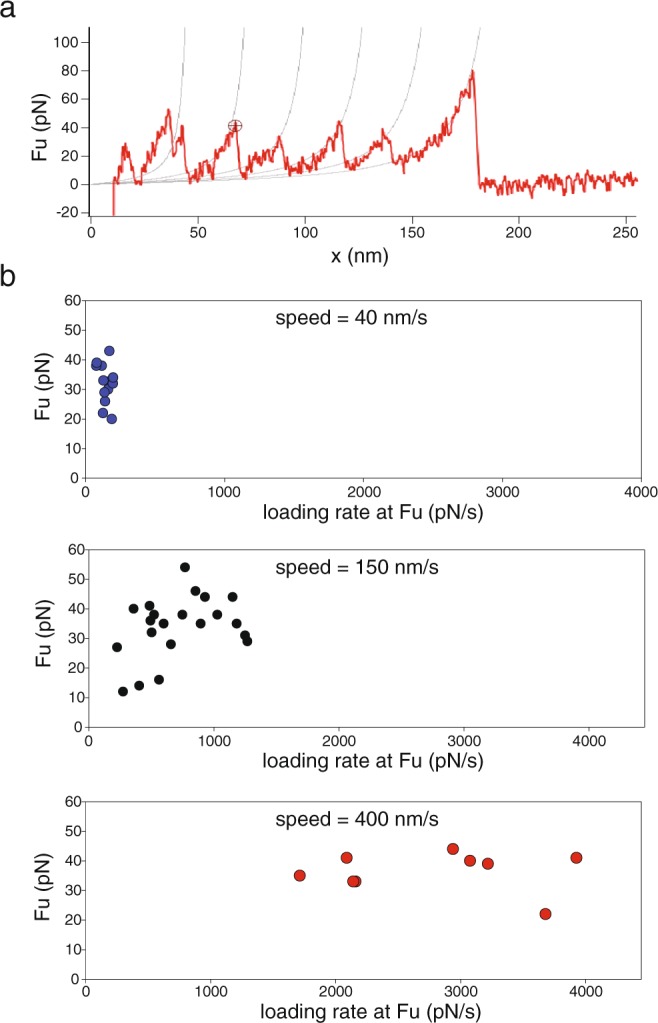
Force extension curves for spectrin unfolding by AFM. (**a**) Representative unfolding trace from one AFM experiment. (**b**) Unfolding forces and loading rates of 32 unfolding events obtained at three different pulling speeds.

To investigate spectrin unfolding at high loading rates we employed single-molecule AFM in a manner similar to the earlier studies of human β-spectrin repeats 1–4^9^ and of chicken brain α-spectrin repeats 15–17^10^ and 13–18^11^. Since all of these studies observed comparable unfolding forces for individual spectrin domains, we expected the AFM unfolding of the human β- spectrin repeats 12–16 studied here to yield similar results. We performed AFM unfolding experiments with the same protein as prepared for the magnetic tweezers experiments above, but now in PBS buffer. The protein was adsorbed nonspecifically to a gold surface and another part of the protein was picked up with the AFM cantilever tip, also via nonspecific adsorption. We then retracted the cantilever at a pulling speeds of 40, 150, or 400 nm/s and measured the force exerted on the cantilever as a function of its distance from the surface. The collected traces showed the sawtooth pattern expected when individual domains unfold stochastically, suggesting that several spectrin repeats were enclosed between the cantilever and the surface (Fig. 5). We then estimated the forces at the moment of unfolding as the distance of the sawtooth maximum to a baseline by fitting the traces to the Wormlike Chain (WLC) model (Fig. 5). We derived the loading rate at each unfolding event using the WLC equation assuming that the elastic behavior observed was due to extension of previously unfolded protein^14,23^. At a pulling speed of 40 nm/s, the mean (+/− standard error) of the unfolding force distribution was 32 +/− 1 pN and the mean of the of the loading rate distribution was 145 +/− 11 pN/s. The corresponding values were 34 +/− 3 pN and 720 +/− 70 pN/s for pulling speeds of 150 nm/s and 38 +/− 2 pN and 2698 +/− 242 pN/s for pulling speeds of 400 nm/s. The measured unfolding forces are well within the range of the unfolding forces of 30 to 50 pN observed in the other AFM studies of spectrin unfolding^9–11^. The variation in the loading rates comes from the fact that they are estimated from the raw data in the force extension curve at each individual unfolding event, which in turn occurs stochastically. The variation is increased by the fact that the spectrin molecules attached through nonspecific interactions with the surfaces of cover slip and cantilever at either end so that (partially) denatured regions add extra spacers that change the protein spring constant and hence the estimated loading rate. The instrumentation itself adds to the variation only in a minor way so that the puling speeds, in contrast to the loading rates, show little variation.

### Loading rate dependence is different in magnetic tweezers and AFM measurements

**Figure 6:**
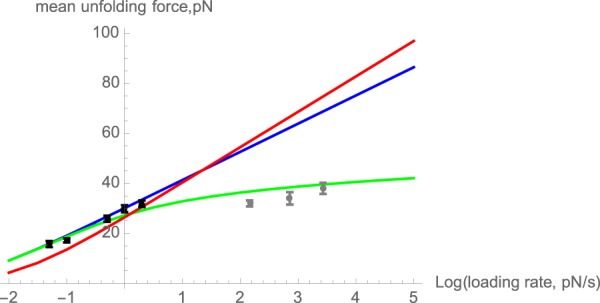
Loading rate dependence of the mean unfolding force. The black markers represent the experimental magnetic tweezers data, and the gray markers represent the AFM data. Blue line and red lines the predictions based on Eqs 1 and 3 with the parameters estimated using Eq. 2 (Model A: blue) and from the location of the distribution peaks (Model B: red). The theoretical mean unfolding force for each model was estimated numerically as the mean of the distribution, Eq. 1, with the *k*(*F*) chosen according to each model. Note that only the magnetic tweezers data were used in estimating the model parameters. The green line gives the predictions of Model A modified to include a sixth order term (Eq. 11) while keeping other parameters unchanged.

The range of the loading rates for which the AFM measurements were made was estimated to be 145–2,700 pN/s. Extrapolation of the magnetic tweezers data from the preceding section to these loading rates using the EZB fit yields a logarithmic dependence of the mean unfolding force on the loading rate and predicts an unfolding force in a range of ~50–75 pN (Fig. 6, blue and red lines), much higher than the measured unfolding forces of below 40 pN (Fig. 6). There are several possible explanations of this discrepancy: (i) Eq. 1 may be invalid, especially at high loading rates. Indeed, Eq. 1 is based on the adiabatic approximation (ref.^33^) where the folded molecule is in local equilibrium at any given value of the force *F*. When this approximation is inadequate, a microscopic model of spectrin dynamics would be required to describe its mechanical response. (ii) Coupling between the molecule and the experimental apparatus may affect the observed unfolding dynamics thus causing discrepancy between the observations made by different experimental techniques. (iii) The force dependence of the unfolding rate *k*(*F*) may be more complex than that described by the EZB model, Eq. 4. More specifically, the loading rate dependence observed in Fig. 6 is indicative of the *anti-Hammond effect*, where the activation length Δ*x* increases with the force *F*. In this case, the barrier Δ*G*(*F*) is no longer a linear function of the force, and Eq. 4 is incorrect. However, the following generalization of Eq. 4, written in a differential form, is still true^22,32,37^:

8 $$d{\rm{\Delta }}G(F)/dF=-\,{\rm{\Delta }}x(F)$$

Since we have $k(F)={k}_{0}{e}^{-\frac{{\rm{\Delta }}G(F)}{{k}_{B}T}}$, Eq. 8 implies that $d\,\mathrm{ln}\,k(F)/dF={({k}_{B}T)}^{-1}{\rm{\Delta }}x(F)$. Substituting this result into Eq. 6, one finds that the formula for the peak force *F*_*m*_, Eq. 7, still holds even for a non-constant activation length Δ*x*. To obtain *F*_*m*_ this equation must now be solved self-consistently, viewing the activation length as a function of this force, Δ*x* = Δ*x*(*F*_*m*_). As a result, the dependence of the peak force on the logarithm of the loading rate, ln *r*, is no longer linear because Δ*x* varies with the value of *r*. This leads to curvature in a plot of *F*_*m*_ vs ln *r*.

To understand the sign of this curvature, it is expedient to think of ln *r* as a function of *F*_*m*_, as in Fig. 4. Differentiating Eq. 7 with respect to *F*_*m*_, one obtains:

9 $$\frac{d\,\mathrm{ln}\,r}{d{F}_{m}}=\frac{{\rm{\Delta }}x}{{k}_{B}T}+\frac{d{\rm{\Delta }}x}{d{F}_{m}}(\frac{{F}_{m}{\rm{\Delta }}x/{k}_{B}T-1}{{\rm{\Delta }}x})$$

For constant Δ*x*, this derivative is constant, given a linear relationship between the peak force and ln *r*. Assuming that the force satisfies the inequality $F{\rm{\Delta }}x\gg {k}_{B}T$ (which is a reasonable expectation since the force would not cause any significant speedup of the unfolding process otherwise), we find that, if Δ*x* decreases with increasing force, the second term in Eq. 9 is negative, and thus ln *r* increases with the force slower than linearly. Equivalently, *F*_*m*_ increases with the loading rate faster than logarithmically. This scenario corresponds to the Hammond effect, where the folded state and the transition state approach one another is the force increases.

In contrast, if the transition state moves away from the native state as the force increases, a scenario known as the anti-Hammond behavior, then the dependence of the peak force on the loading rate is weaker than logarithmic, which is the case for our data (Fig. 6). We note that, although the above arguments describe the relationship between the peak (rather than mean) force and the loading rate, the mean force shown in Fig. 6 behaves in a similar way.

Generally speaking, the anti-Hammond effect is caused by a multi-dimensionality of the underlying energy landscape^18,37–39^ and may arise in the cases of both single and multiple unfolding pathways. For a single unfolding pathway, the simplest model that accounts for both the Hammond and anti-Hammond effects is the force Taylor expansion of the free energy barrier assuming sufficiently low force [see, e.g., Konda *et al*.^37^]:

10 $${\rm{\Delta }}G(F)=-\,{k}_{B}T\,\mathrm{ln}\,k(F)/{k}_{0}\simeq {\rm{\Delta }}G(0)-F{\rm{\Delta }}x-{F}^{2}{\rm{\Delta }}\chi /2$$

The first term is the same as in EZB, but the second term now accounts for the force-induced movement of both the native (N) and the transition (TS) states according to the equation $d{x}_{TS(N)}/dF={\chi }_{TS(N)}$, with ${\rm{\Delta }}\chi ={\chi }_{TS}-{\chi }_{N}$ being the change in the compliance (i.e., the inverse stiffness) from the native to the transition state. The sign of Δ*χ* determines whether Hammond or anti-Hammond behavior is observed. A positive value of Δ*χ* implies that the transition state has positive compliance and that it is more compliant than the folded state; as a result, the location of the transition state moves away from that of the native state as the force is increased, resulting in an increasing value of the activation length Δ*x* - this is the anti-Hammond behavior. A positive value of $\Delta \chi$ further implies a faster than exponential increase of the rate with the force because the barrier decreases with the force faster than linearly. It is easy to see that this results in the mean unfolding force rising with ln*r* slower than linearly, as in Fig. 6. Indeed, at any given loading rate *r* the protein will tend to unfold sooner than in the case of the exponential *k*(*F*), resulting in a lower mean unfolding force and thus weaker dependence of the mean unfolding force on ln*r* (which is close to linear for the exponential *k*(*F*)).

Can inclusion of the quadratic term, as in Eq. 10, account for the experimental loading rate dependence seen in Fig. 6? Fitting the magnetic tweezers data using Eq. 10 amounts to fitting ln *k*(*F*) to a second-order polynomial in *F*, which would allow for curvature of the fits of Figs 3 and 4. Unfortunately, doing so does not result in statistically significant improvement of the fits (although it is curious that the value of Δ*χ* was found to be positive for both Model A and Model B, which corresponds to anti-Hammond behavior). More importantly, the rather small values of Δ*χ* that are consistent with the lack of curvature observed in Figs 3 and 4 (Δχ ≤ 0.01 nm/pN) do not produce a significant effect when extrapolated to the AFM force range. In other words, it is impossible to reconcile the magnetic tweezers and the AFM data by merely assuming the quadratic dependence of the form of Eq. 10.

Instead, our data suggest a much stronger anti-Hammond effect than that estimated when only a quadratic correction to the force dependence of the free energy is included. Such stronger anti-Hammond behavior may arise from higher order terms, which are neglected in the expansion of Eq. 10, which, as a result, would only be appropriate for short extrapolations. For extrapolations of the unfolding rate constant *k*(*F*) over longer force (*F*) ranges, it may be necessary to include higher order terms in Eq. 10. To illustrate the effect of higher-order terms, consider a modified model that includes an additional sixth order term,

11 $$k(F)={k}_{0}{e}^{aF+c{F}^{6}},$$

where the coefficients *a*, and *k*_0_ are the same as in Model A and *c* is a newly introduced constant that does not have an immediate physical meaning. Inclusion of this higher-order term (with $c=2.0\times {10}^{-9}\,p{N}^{-6}$) brings model A much closer to the experimental data in the entire range of forces examined (Fig. 6 green line).

There is, of course, no physical reason to include specifically a sixth order term, and very similar results are obtained with a fourth or eight order term (with an appropriate coefficient) as well as with a combination of such higher order terms. In order to agree with both Model A at low force and with the high force AFM data, any such model must possess two features: First, the higher order terms must be negligible in low loading rate regime explored by the magnetic tweezers experiments, and, second, they should be *positive* resulting in a faster than exponential growth of *K*(*F*) at high forces and, consequently, in lower unfolding forces.

## Discussion

We have investigated the force-induced unfolding kinetics of spectrin repeats over a wide range of unfolding forces and loading rates by combining magnetic tweezers experiments with traditional AFM experiments. In contrast to the results reported by high-force AFM studies, which demonstrate only a weak dependence of the unfolding rate on force^9–11^, the results from magnetic tweezers experiments at low unfolding forces reveal a much stronger dependence. Indeed, within an experimental range of forces of 10–40 pN the unfolding rate varies over nearly 2 orders of magnitude (Fig. 3). Consequently, the mean unfolding force changed by a factor of ~2 as the loading rate was increased over a factor of 40 (Fig. 6). These observations are consistent with a roughly exponential increase of the unfolding rate with the applied force, which, in turn, corresponds to the unfolding barrier decreasing linearly with force, ${\rm{\Delta }}G\approx {\rm{\Delta }}G(0)-F{\rm{\Delta }}x$, as in the commonly accepted Eyring-Zhurkov-Bell (EZB) formula. The value of the activation length Δ*x* (i.e., the amount by which the protein is elongated before it reaches the unfolding transition state) is deduced from these results to be in a range Δ*x* ≈ 0.65–0.85 nm, comparable to values found for other proteins^40^.

When the EZB predictions are extrapolated to higher loading rates corresponding to the regime probed by AFM, however, we find that they overestimate the average unfolding force and its loading rate dependence, as compared to the actual AFM data reported here (as well as the published AFM for slightly different spectrin constructs). In terms of the EZB model, the weaker loading rate dependence found in the AFM measurements can be interpreted as a longer activation length, suggesting that the activation length increases with an increasing loading rate/unfolding force.

There are several possible causes of this discrepancy:

(i) It is conceivable that the model where the probability of unfolding obeys first-order kinetics with a force- (and thus time-) dependent rate *k*(*F*) breaks down at high forces, as this model assumes that the force *F* changes slowly enough that quasi-equilibrium at each value of the force exists^33^.
(ii) It is also conceivable that the disagreement between the magnetic tweezers and AFM data seen in Fig. 6 is due to instrumental differences (e.g., force calibration errors, etc.). One further caveat that should be considered when combining magnetic tweezers data with AFM data is the influence of the instrument on the measured unfolding rate. Because in both experimental techniques the protein is elastically coupled to a much larger probe (AFM tip/cantilever assembly or magnetic bead), which is subjected to considerable hydrodynamic drag, the observed unfolding rate may be different from the intrinsic unfolding rate, and it may thus be different for different techniques. The effect of the instrument on the observed rate has been the subject of recent interest^41–44^, with the conclusion that this effect can be complicated and dependent on the elasticity of the linker connecting the protein and the probe and the hydrodynamic drag on the probe. While we cannot rule out that the discrepancy between the magnetic tweezers and AFM data is caused by differences in the experimental techniques, we note that the above studies suggest that the effects of the instrument on the unfolding dynamics is to change the prefactor *A* in the Arrhenius/Kramers equation, $k(F)=A{e}^{-\frac{{\rm{\Delta }}G(F)}{{k}_{B}T}}$, by modifying the effective friction along the reaction coordinate. It seems less likely that such experimental differences would affect the observed activation length Δ*x* thereby changing the *slope* of the loading rate dependence (cf. Eq. 9), which is observed to become weaker at higher loading rates/forces.
(iii) Finally, the EZB-predicted linear force dependence of the activation barrier (Eq. 4) may only be applicable in a limited range of forces probed by a single experimental technique. Indeed, more accurate theories predict a nonlinear force dependence of the free energy barrier^38^. Depending on how the activation length depends on the applied force, one can differentiate between Hammond behavior^45^, where it decreases with the force, and anti-Hammond behavior, where it increases^17,18,46^. Hammond behavior is more familiar, has been predicted by simple one-dimensional kinetic models and is observed in the chemical unfolding of several proteins^47–50^. Anti-Hammond behavior is a manifestation of essential multidimensionality of the underlying free energy landscape^37,39,51–53^.

The data reported here and obtained by two different experimental techniques is consistent with anti-Hammond behavior: the unfolding behavior at low forces, as investigated with magnetic tweezers, can be reconciled with the behavior at higher forces, as investigated in the AFM experiments, if the activation length increases with increasing force. Indeed, extrapolation of the logarithmic dependence of the mean unfolding force 〈*F*〉 on the loading rate *r* in the magnetic tweezer experiments to the values of *r* in the AFM regime would predict much higher unfolding forces than those observed experimentally, indicating a slowdown in the 〈*F*〉 vs ln *r* dependence (Fig. 6). Such a change in the dependence of the unfolding force on the loading rate is not compatible with the EZB model, which predicts the slope of this dependence to be constant and inversely proportional to the activation length^32^,

12 $$d\langle F\rangle /d\,\mathrm{ln}\,r\simeq \frac{{k}_{B}T}{{\rm{\Delta }}x},$$

A decrease in the slope with the increasing force implies that the activation length must increase (note that Eq. 12 immediately follows from Eq. 9 if one assumes force-independent activation length and neglects the difference between the peak force *F*_*m*_ and its average value *F*). We note that significant deviations from the simple logarithmic increase of the mean unfolding (or unbinding) force with the loading rate have been reported in other studies (e.g.^54–56^). In contrast to our results, however, these studies observed an opposite trend; specifically, the mean unfolding force exhibited a biphasic behavior, with slow logarithmic increase at low loading rates superseded by fast logarithmic increase at high loading rates. This behavior is consistent with the Hammond effect and was interpreted as evidence of complexity of the unfolding/unbinding kinetics. Here we find change in the opposite direction, thus an anti-Hammond effect.

The anti-Hammond effect necessitates that the unfolding rate increases faster than exponentially as the pulling force increases. This behavior follows from the exact relationship (Eq. 8) between the dependence of the free energy barrier on the applied force and the (force-dependent) activation length^22,57^,

13 $$d\,\mathrm{ln}\,k(F)/dF\approx -\,{({k}_{B}T)}^{-1}d{\rm{\Delta }}G/dF={\rm{\Delta }}x/{k}_{B}T$$

The slope of the dependence ln *k*(*F*) on force will increase if Δ*x* also increases with the force. The observation of anti-Hammond behavior highlights limitations of the simple, one-dimensional models often invoked to explain force induced unfolding kinetics. Such models consider the protein’s free energy *G*(*x*) viewed as a function its extension *x*; the native state then corresponds to a minimum *x*_*N*_ of *G*(*x*). When subjected to a force, the location of this minimum is shifted in the direction of the force, consistent with the common experience that objects are elongated rather than shrunk in response to stretching forces. Conversely, the position of the transition-state *x*_*TS*_ corresponds to a maximum of *G*(*x*); as a result, it is shifted in the direction opposite to the force because the barrier curvature (effectively playing the role of the spring constant) is negative. Provided that the activation length, ${\rm{\Delta }}x={x}_{TS}-{x}_{N}$, is positive, the transition state moves toward the folded state inevitably resulting in Hammond rather than anti-Hammond behavior.

Anti-Hammond behavior can result from different physical mechanisms. One possibility is that it results from a switch in the unfolding pathway as the force changes^52^. In this scenario unfolding can occur through two parallel pathways, and the barrier to unfolding by force for each pathway obeys the simple EZB relationships, ${\rm{\Delta }}{G}_{1}(F)={\rm{\Delta }}{G}_{1}(0)-F{\rm{\Delta }}{x}_{1}$, and ${\rm{\Delta }}{G}_{2}(F)={\rm{\Delta }}{G}_{2}(0)-F{\rm{\Delta }}{x}_{2}$. If ${\rm{\Delta }}{G}_{1}(0) < {\rm{\Delta }}{G}_{2}(0)$ and ${\rm{\Delta }}{x}_{1} < {\rm{\Delta }}{x}_{2}$, then the first pathway is dominant (i.e. has a lower barrier) at low forces. However, the longer activation length of the second pathway causes its barrier to unfolding, ${\rm{\Delta }}{G}_{2}(F)$, to decrease faster with the force increase, so that it may become dominant at high forces. As a result, the activation length equals Δ*x*_1_ at low force but attains a higher value Δ*x*_2_ when the force is raised, which is the anti-Hammond effect. Indeed, this type of scenario was invoked to explain experimental measurements by Jagannathan *et al*.^16^. A second mechanism leading to the anti-Hammond effect exists even in a single-pathway scenario and is a fundamental consequence of the multi-dimensionality of the underlying free energy landscape^17,58^. On a multidimensional free energy landscape, the transition state is no longer a maximum but instead a saddle point, with some directions leading uphill in free energy and others downhill. Unlike in the one-dimensional model discussed above, then, the transition-state compliance *χ*_*TS*_ (inverse of spring constant) is negative only if the pulling coordinate *x* happens to be well aligned with the “intrinsic” unfolding coordinate that corresponds to the downhill direction so that the two coordinates are essentially the same. In the absence of such alignment, the transition-state compliance is positive and, if the transition state is further softer than the native state (i.e., *χ*_*TS*_ > *χ*_*N*_) then anti-Hammond effect ensues. For a free energy landscape of a high dimensionality, there are many directions leading uphill from the transition state but only one leading downhill (corresponding to the intrinsic unfolding coordinate). It was thus argued^17^ that, if the underlying dimensionality of the free energy landscape is sufficiently high, low force behavior of the transition-state will almost universally exhibit anti-Hammond rather than Hammond effect. Although a single-pathway unfolding mechanism^17,58^ is sufficient to describe the anti-Hammond effect observed in our measurements, the data described here do not allow us to discriminate between single- and multiple-pathways mechanisms.

Despite theoretical arguments and simulations that support the anti-Hammond effect^18,21^, prior experimental evidence of the anti-Hammond behavior is limited, to our knowledge, to a single report^16^. There are several likely reasons for the dearth of experimental support of this effect. Specifically, the anti-Hammond effect is expected to become superseded by Hammond behavior at sufficiently high forces, when the intrinsic unfolding coordinate becomes aligned with the pulling coordinate^58^. Low-force measurements are often limited by instrument force stability and by the timescale of the process, which may be too slow to observe at low forces. In addition, measurement of the curvature in the ln *k*(*F*) dependence on force needed to distinguish between Hammond and anti-Hammond behavior requires accurate measurements in a sufficiently broad range of forces that are difficult to accomplish without combining different techniques. This type of study may become more common with the advances in single-molecule force spectroscopy^59^.

## Conclusions

We report a single molecule force spectroscopy study of the mechanically induced unfolding of spectrin that combines low-force measurements by magnetic tweezers with high force measurements by AFM. The unfolding forces that we have probed thus range from a few piconewtons, which is comparable to the forces occurring *in vivo*, to ~50 piconewtons. The data reveal that the unfolding rate is much more sensitive to the force in the high force regime probed by AFM than in the low force regime probed by magnetic tweezers. This behavior is consistent with the anti-Hammond effect, a phenomenon that is intrinsically linked to multi-dimensionality of the underlying free energy landscape where the unfolding transition state moves away from the native state as the applied mechanical force increases. While predicted to be universal by theoretical studies, this behavior is rarely observed and contrary to the commonly used one-dimensional models of single pathway protein unfolding. The difference between the behavior observed by two different experimental techniques may alternatively be caused by inadequacy of the theoretical model of simple first-order kinetics used in the data analysis or by dynamical effects that are imposed on molecular dynamics by the AFM and tweezer setups. Definitive explanation of the difference in the behavior observed by the two techniques would thus require further experiments using improved single-molecule techniques capable of bridging the force gap between the two techniques.

## Methods

### Protein construction and preparation

We cloned the human β-spectrin repeats 12–15 flanked by a 6xHis tag and a SNAP domain at the N-terminus and a 23 amino acid biotinylation tag at the C-terminus in the first multiple cloning site of the protein expression plasmid pETduet. The same plasmid expressed the biotin protein ligase BirA in the second multiple cloning site so that both proteins would be made in the same cell and BirA could biotinylate the 23 amino acid C-terminal tag of the spectrin construct. The plasmid was transformed into the Rosetta (DE3) *E. coli* strain. Cells were grown in 2 L LB supplemented with 100 μg/mL of ampicillin and 50 μg/mL biotin to an OD_600_ ~ 0.5 and protein expression was induced by 0.5 mM IPTG for 3 hours at 28 °C. Cells were lysed by 2 passes at 15,000 PSI through an Emulsiflex C3 homogenizer (Avestin). The clarified lysate was passed over a 5 mL NTA column. The eluted NTA fractions were concentrated and passed over a 16/60-superdex S200 size exclusion column. Purity was assessed by SDS-PAGE followed by Coomassie staining and UV-vis absorbance. Pure fractions were pooled and stored at −80 °C in PBS, 10% glycerol and 2 mM DTT.

### Flow cell assembly and functionalization

Flow cells were constructed by heating strips of parafilm between a silanated coverslip and microscope slide, with holes in the slide made and the parafilm cut to define four separate flow channels per slide. The channels were washed with 100 mM Sodium Bicarbonate buffer pH 8.5. A solution of 250 μg/μL of BG-PEG-NH_2_ (New England Biolabs) and amino microspheres (Polysciences, 1:200 dilution) in 100 mM Sodium Bicarbonate buffer pH 8.5 was incubated with the coverslip surface for 3 hours. Non-magnetic amino microspheres (3 micron diameter, Polysciences, <8% coefficient of variance in diameter) were used as reference beads to eliminate drift. The unbound ligand and beads were washed out with 500 μL of 1 M Tris pH 8.0 and channels were incubated for 30 minutes to block unreacted epoxide groups. Channels were then washed with 500 μL of buffer containing 10 mg/mL BSA, PBS, 2 mM DTT and 0.01% tween and blocked with BSA overnight at room temperature to prevent non-specific binding of beads to the surface. Additionally, the streptavidin-coated magnetic beads (2.7 µm diameter, M-270 Strepavidin, ThermoFisher, <3% coefficient of variance in diameter) were also blocked against non-specific binding by diluting 10 μL of streptavadin beads into 300 μL of 10 mg/mL BSA, PBS, 2 mM DTT and 0.01% tween and incubated overnight at 4 °C while gently rocking to prevent the beads from settling.

### Force calibration for magnetic tweezers

The force calibration was performed as described previously^15,60^, based on the Brownian calibration scheme used widely in magnetic tweezers experiments, namely that pulling force is inversely related to transverse fluctuations. This is straightforward to implement for tethered DNA molecules that are sufficiently long (3,000 nm) that the transverse bead fluctuations are quite large even for high (100 pN) forces^23^. However, for the much shorter protein tethers (100 nm) the fluctuations are small so that direct calibration is possible only at low forces (<5 pN); this is a problem since there is appreciable variation in pulling force from bead to bead. We solved this problem using the approach described in ref.^23^, whereby a data set on a long DNA tether was collected to provide a calibration of force versus magnet position. For each short protein tether studied, a series of transverse fluctuation recordings were collected at 3 to 5 different magnet positions below 5 pN, where the fluctuations were large enough to be measurable. These measurements were used to determine a tether-specific amplitude that was applied to the calibration curve to obtain the force versus magnet position curve for that short protein tether, as described in ref.^23^.

### Constant loading-force magnetic tweezers experiments

To determine the relationship between magnet position and force of a tethered bead we fit the magnet position vs. ln(force) curves of five tethered beads to a polynomial and obtained a function describing this relationship. This function was then used to move the magnet at the appropriate rate to achieve the desired linear loading rate.

### Atomic-force-microscopy (AFM) experiments

Pulling experiments using AFM were carried out on a Cypher AFM instrument from Asylum Research and on a home-built single-molecule AFM as previously described in the literature^60^. In each experiment, we deposited 1 μL of spectrin solution (0.5 mg/mL) in PBS onto a glass substrate covered by 50 μL of PBS. We allowed the protein to absorb onto the substrate for 10 minutes before pulling. The spring constant of each individual cantilever (MLCT or Olympus OBL, Bruker AFM Probes, Camarillo, CA) was determined experimentally by measuring the cantilever power spectrum and using the equipartition theorem^61^ at the beginning of each experiment.

## Supplementary information

Supplementary information

## Data Availability

The raw datasets generated during and analyzed in the current study are available from the corresponding authors on reasonable request.
