# Supplementary material for: Mechanical unfolding of spectrin reveals a super-exponential dependence of unfolding rate on force
Source: Sci Rep. 2019 Jul 31;9:11101. doi: 10.1038/s41598-019-46525-w (PMC6668576; doi:10.1038/s41598-019-46525-w)
Supplement: Supplementary file 1 — Supplementary information [file 41598_2019_46525_MOESM1_ESM.pdf]

Supporting information for

Mechanical unfolding of spectrin reveals a super-exponential dependence of unfolding rate on force

J. P. Renn, S. Bhattacharyya, H. Bai, C. He, H. Li, A.F. Oberhauser, J. F. Marko, D. E. Makarov and A. Matouschek

SUPPLEMENTARY METHODS

Cleaning and silanization of coverslips

Coverslips and microscope slides were first rinsed with many times with water to remove debris from the packaging and then cleaned in a 2% Hellmanax solution for 15 minuets. The coverslips and slides were rinsed with water and further cleaned and etched by sonicated in 1 M KOH for 30 minuets. The washed and etched slides and coverslips were rinsed many times with water and then stored in 100% ethanol until silanization. The coverslips were reacted with a solution of 2% (3-Glycidyloxypropyl)trimethoxysilane (Sigma Aldrich 440167) in ethanol for 30 min. The reacted coverslips were washed 3 times with 100% ethanol to remove the unbound silane and the coverslips were incubated for 30 min at 100 degrees C to cure the silane.

Testing the surfaces

Flow cell channels were washed with 100 mM Sodium Bicarbonate buffer pH 8.5. A solution of 250 µg per µL of BG-PEG-NH2 and amino microspheres (1:200 dilution) in 100 mM Sodium Bicarbonate buffer pH 8.5 was incubated with the coverslip surface for 3 hours. The unbound ligand and reference beads were washed out with 500 µL of 1 M Tris pH 8.0 and incubated for 30 minutes to block unreacted epoxide groups. Channels were then washed with 500 µL of buffer containing 10 mg/mL BSA, PBS, 2 mM DTT and 0.01% tween and blocked overnight at room temperature to prevent non-specific binding of beads to the surface. Additionally the streptavidin coated magnetic beads were also blocked against non-specific binding by diluting 10 uL of M270 streptavadin beads into 300 µL of 10 mg/mL BSA, PBS, 2 mM DTT and 0.01% tween and incubated overnight at 4 ˚C while gently rocking to prevent the beads from settling. We needed to make surfaces that prevented non-specific binding of the beads and proteins and that specifically bound the SNAP domain for specific linkage to the magnetic bead. To test our surfaces we incubated the surface with protein or buffer and then beads and counted the number of beads bound to the surface. When we have no protein incubated in the channel and only beads we see very few beads bound to the surface. Then as we increase the amount of protein we see more beads bound to the surface. We know this linkage is specific because when we use a construct that is lacking the C-terminal biotin tag we see very few beads bound.

Finding tethered beads

One nM SNAP-spectrin was bound to the surface for 20 min and washed out with 500 µL of buffer containing 10 mg/mL BSA, PBS, 2 mM DTT and 0.01% tween. Twenty µL of blocked M270 streptavadin coated magnetic beads were flowed into the channel and incubated with the surface for 20 min. Unbound beads were washed out with buffer. The channels were checked by looking at the number for beads bound when no SNAP-spectrin was incubated in the flow channel. Generally there were very few beads bound to the surface when no protein was incubated in the channel.

Data analysis

Transitions were identified in the extension vs. time data by inspection and a region extending from approximately three seconds before the transition to approximately three seconds after the transition were extracted for further analysis. The extracted data were fit to a Heaviside step function (red circles, Figure SI 5) and the change in extension (ΔZ) was taken as the difference between horizontal baselines fitted to the datapoints before and after the transition (black arrows, Figure SI 5). The released contour length was then calculated using Wormlike Chain equation and the change in extension during the transition, as described previously (31). We set the unfolding force for a specific transition as the mean of

the forces determined for the magnet position corresponding to the baselines preceding and following the transition. Generally the change in magnet position across a transition was too small to result in a change in force above experimental error (i.e. the force at the beginning and end of the transition were the same).

SUPPLEMENTARY FIGURES

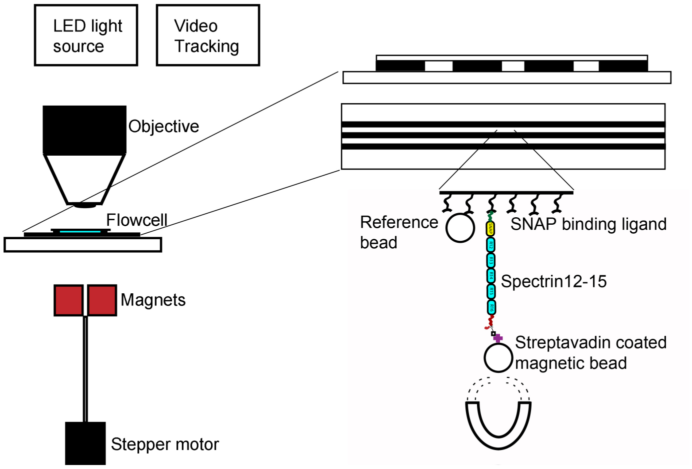

Figure SI 1: Experimental set up. Schematic drawing of the magnetic tweezers set up (left) and the tethering scheme (right). The protein is covalently attached to the functionalized surface via an N-terminal SNAP domain and then a streptavidin coated magnetic bead is attached to a biotin moiety at the C terminus of the protein to tether a magnetic bead to the surface via a the spectrin construct spanning five spectrin domains. Non-magnetic reference beads are attached to the surface to prevent drift since high forces are used in these experiments. An inverted light microscope monitors the position of the beads in real time while magnets below the objective generate force on the tethered beads. A stepper motor controls the magnet position and moving the magnets closer to the stage generates more force on the bead while moving away lowers the force.

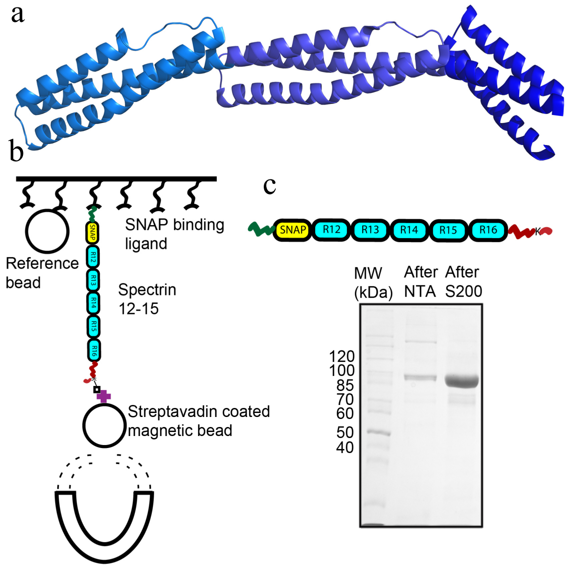

Figure SI 2: (a) Crystal structure of human erythroid β-spectrin repeats 13-15 (PDB: 3KBT). (b) Schematic construct used in these studies. (c) SDS-PAGE analysis of the purified chimeric protein.

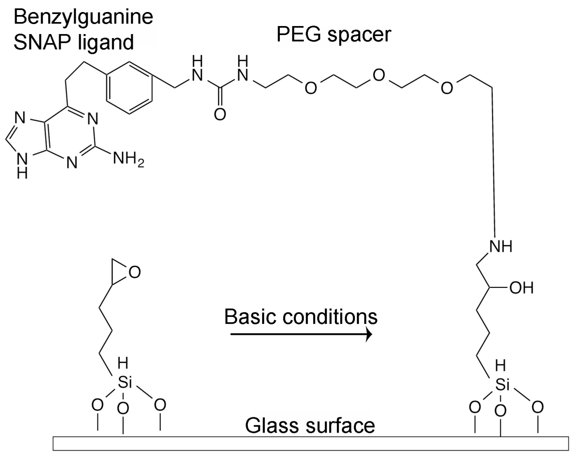

Figure SI 3: Schematics of the reaction to modify the glass coverslips to specifically bind the SNAP protein. An amino reactive epoxide silane was applied to cleaned coverslips. Then under basic conditions the SNAP ligand with a PEG spacer and reactive amine handle was reacted to the epoxide groups on the surface to form a covalent linkage between the surface and the SNAP binding ligand.

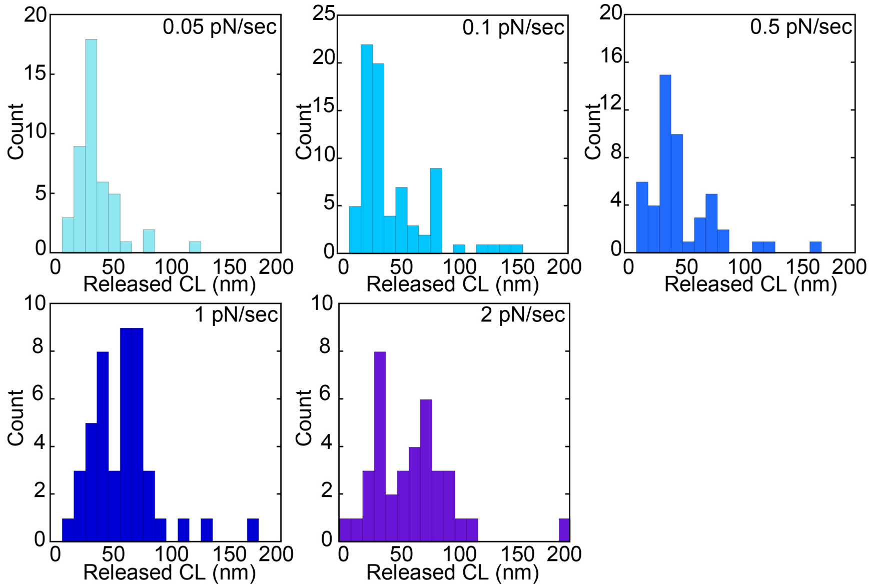

Figure SI 4: The effect of loading rate on spectrin unfolding contour length. The contour lengths of each spectrin transition was measured and calculated at loading rates of 0.05, 0.1, 0.5, 1 and 2 pN s-1, binned and plotted in a histogram (n = 41, 77, 49, 47, and 38 respectively).

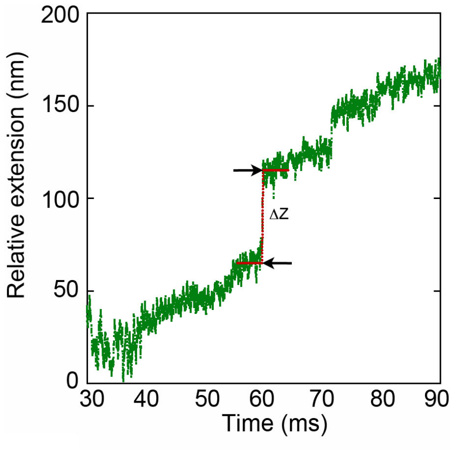

Figure SI 5: Step fitting of the extension vs. time data. A sample transition is selected and then fit with a Heaviside step function (red circles). The flat portions at the beginning and the end of the transition are indicated with black arrows. The change in extension (ΔZ) is the difference between the flat portions before and after.
